# Theoretical Study of the Antioxidant Activity of Quercetin Oxidation Products

**DOI:** 10.3389/fchem.2019.00818

**Published:** 2019-11-27

**Authors:** Alejandro Vásquez-Espinal, Osvaldo Yañez, Edison Osorio, Carlos Areche, Olimpo García-Beltrán, Lina María Ruiz, Bruce K. Cassels, William Tiznado

**Affiliations:** ^1^Computational and Theoretical Chemistry Group, Departamento de Ciencias Químicas, Facultad de Ciencias Exactas, Universidad Andres Bello, Santiago, Chile; ^2^Facultad de Ciencias Naturales y Matemáticas, Universidad de Ibagué, Ibagué, Colombia; ^3^Departamento de Química, Facultad de Ciencias, Universidad de Chile, Santiago, Chile; ^4^Facultad Ciencias de la Salud, Instituto de Ciencias Biomédicas, Universidad Autónoma de Chile, Santiago, Chile

**Keywords:** antioxidant, quercetin, DFT calculations, oxidized derivatives of quercetin, molecular docking

## Abstract

It was recently shown that, when tested in cellular systems, quercetin oxidized products (Qox) have significantly better antioxidant activity than quercetin **(Q)** itself. The main Qox identified in the experiments are either 2,5,7,3′,4′-pentahydroxy-3,4-flavandione **(Fl)** or its tautomer, 2-(3,4-dihydroxybenzoyl)-2,4,6-trihydroxy-3(2*H*)-benzofuranone **(Bf)**. We have now performed a theoretical evaluation of different physicochemical properties using density functional theory (DFT) calculations on **Q** and its main Qox species. The most stable structures (for **Q** and Qox) were identified after a structural search on their potential energy surface. Since proton affinities (PAs) are much lower than the bond dissociation enthalpies (BDEs) of phenolic hydrogens, we consider that direct antioxidant activity in these species is mainly due to the sequential proton loss electron transfer (SPLET) mechanism. Moreover, our kinetic studies, according to transition state theory, show that **Q** is more favored by this mechanism. However, Qox have lower PAs than **Q**, suggesting that antioxidant activity by the SPLET mechanism should be a result of a balance between proclivity to transfer protons (which favors Qox) and the reaction kinetics of the conjugated base in the sequential electron transfer mechanism (which favors **Q**). Therefore, our results support the idea that **Q** is a better direct antioxidant than its oxidized derivatives due to its kinetically favored SPLET reactions. Moreover, our molecular docking calculations indicate a stabilizing interaction between either **Q** or Qox and the kelch-like ECH-associated protein-1 (Keap1), in the nuclear factor erythroid 2-related factor 2 (Nrf2)-binding site. This should favor the release of the Nrf2 factor, the master regulator of anti-oxidative responses, promoting the expression of the antioxidant responsive element (ARE)-dependent genes. Interestingly, the computed Keap1-metabolite interaction energy is most favored for the **Bf** compound, which in turn is the most stable oxidized tautomer, according to their computed energies. These results provide further support for the hypothesis that Qox species may be better indirect antioxidants than **Q**, reducing reactive oxygen species in animal cells by activating endogenous antioxidants.

## Introduction

Flavonoids are a large group of natural polyphenols that are biosynthesized in all “land plants” or embryophytes, and in some possibly ancestral fresh-water green algae (Charophyta). They are therefore present in plant resources normally consumed by humans (Pietta, 2000; Mabry et al., 2012). Among the flavonoids, quercetin (**Q**) (3,5,7,3′,4′-pentahydroxyflavone) is the main representative of the flavonol subclass. **Q** has been shown to have a broad variety of biological activities and pharmacological actions. These properties are largely attributed to quercetin's ability to diminish the formation of reactive oxygen species (ROS) through different mechanisms (Hollman et al., 1997; Pietta, 2000; Pedrielli et al., 2001; Zhang et al., 2011; Mabry et al., 2012; Osorio et al., 2013; Gupta et al., 2016). However, its possible role in numerous prooxidant effects in intracellular environments is also being continually updated (Laughton et al., 1989; Metodiewa et al., 1999; Choi et al., 2003; Kessler et al., 2003; Spencer et al., 2003; Ruiz et al., 2015).

The oxidation processes of **Q**, whether chemically, electrochemically or enzymatically induced, have been widely studied in the literature (Jørgensen et al., 1998; Krishnamachari et al., 2002; Makris and Rossiter, 2002; Brett and Ghica, 2003; Kubo et al., 2004; Zenkevich et al., 2007; Zhou and Sadik, 2008; Barnes et al., 2013). However, there are only a few studies related to the antioxidant properties of the oxidation products of this flavonol (Krishnamachari et al., 2002; Ramos et al., 2006; Gülşen et al., 2007; Fuentes et al., 2017). For example, Gülşen et al. (2007) found that one of its oxidation products, the quercetin heterodimer (QHD), which can also be generated by radical oxidation, is less active than **Q**. On the other hand, Ramos et al. (2006) evaluated the antioxidant activity in the 2,2-diphenyl-1-picrylhydrazyl (DPPH) assay, concluding that some of the oxidation products of **Q** are more active than **Q** itself. More recently, Atala et al. (2017) through a cell-free system assay showed that a quercetin-free metabolite mixture of oxidized quercetin derivatives largely retains the antioxidant activity of the unoxidized flavonoid. However, none of the components of the mixture exceed the antioxidant activity of **Q**. Additionally, Fuentes et al. (2017) evaluated the antioxidant capacity of this mixture, and of each one of its main components, in human skin fibroblast Hs68 and human colon epithelial Caco2 cells undergoing oxidative stress, induced by indomethacin or hydrogen peroxide. This study showed, for the first time, that when tested in a cellular system, the mixture of the oxidation products of **Q** and one of its components show an antioxidant capacity that far exceeds that of **Q**. This paper focuses on explaining this seemingly paradoxical observation by providing some insight into the antioxidant capacity of both **Q** and its main oxidized derivatives from a molecular point of view, using density functional theory (DFT) calculations.

**Figure 1:**
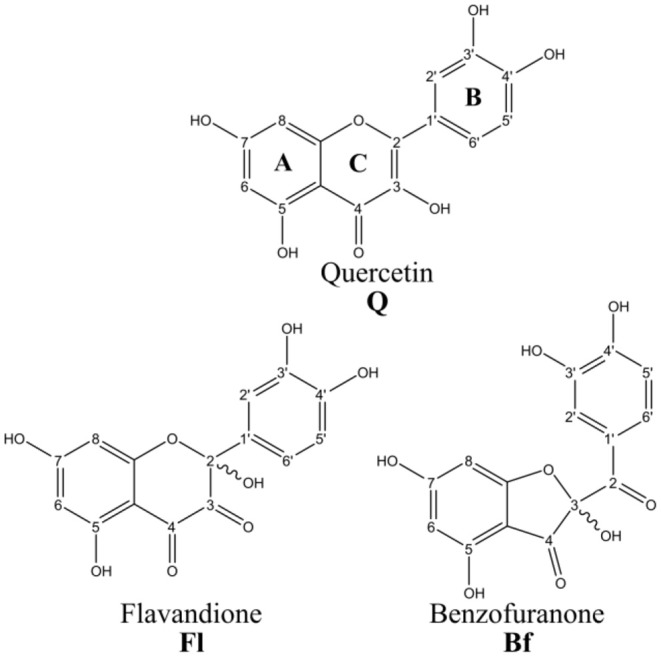
Quercetin and its main oxidized derivatives.

This research will focus on the major quercetin oxidation products (Qox) (Fuentes et al., 2017): 2,5,7,3′,4′-pentahydroxy-3,4-flavandione (**Fl**) and 2-(3,4-dihydroxybenzoyl)-2,4,6-trihydroxy-3(2*H*)-benzofuranone (**Bf**), see Figure 1. The mixture of these two metabolites proved to be an antioxidant at least 200-fd more potent **Q** in an intracellular environment (Fuentes et al., 2017). It is important to mention that **Fl** and **Bf** are isomers interconverting through a ring-chain tautomeric equilibrium.

In general, it is well documented that **Q** confers antioxidant protection to cells by both direct (Bors et al., 1990, 1994; Rice-Evans et al., 1996) and indirect mechanisms (Warwick et al., 2012; Dinkova-Kostova and Abramov, 2015; Messer et al., 2015). While direct mechanisms are related to the participation of **Q** in redox reactions that directly scavenge free radicals, indirect antioxidants exert their activity up-regulating cytoprotective proteins, also known as the “ultimate antioxidants” (Di Meo et al., 2013). In this work, the possible role of **Q**, **Fl**, and **Bf** as both direct and indirect antioxidants was evaluated.

As shown by Di Meo et al. (2013) **Q** can act as a direct antioxidant through two main mechanisms involving any of its phenolic OH groups (ArOH):

Hydrogen atom transfer (HAT):

(1) $$\begin{array}{r} ArOH+R^{\cdot }\to ArO^{\cdot }+RH \end{array}$$

Sequential proton loss electron transfer (SPLET):

(2) $$\begin{array}{r} ArOH↔ArO^{-}+H^{+} \end{array}$$

(3) $$\begin{array}{r} ArO^{-}+R^{\cdot }\to ArO^{\cdot }+R^{-} \end{array}$$

(4) $$\begin{array}{r} R^{-}+H^{+}\to RH \end{array}$$

There is a third mechanism, known as electron transfer-proton transfer (ET-PT), however it can be considered as a hydrogen atom transfer (HAT) due to the very fast proton transfer step. In addition, the first step in this mechanism, electron transfer from the neutral ArOH system to the free radical, is highly unfavorable due to the high instability of the ArOH^.+^ radical cation resulting in too high energy barriers and very low rate constants, which makes ET-PT not competitive with HAT and SPLET mechanisms (Di Meo et al., 2013). Therefore ET-PT mechanism was not considered in this work.

It is important to mention that while the HAT mechanism is more important in non-polar solvents, the SPLET mechanism is favored in polar solvents, since these can stabilize the involved ionic species by solvation. In addition, although in principle both mechanisms have the same thermodynamic balance, the pH and acidity of the molecule are factors that must be taken into account when analyzing antioxidant activity. For instance, at physiological pH (pH = 7.4), about 92% of **Q** is present in its neutral form, and 8% in its deprotonated form, which could considerably tip the balance in favor of the SPLET mechanism. These estimates are made considering that the reported pK_a_ value of **Q** is 8.45 (Musialik et al., 2009).

A common approach for evaluating antioxidant activity by the mechanisms described above, is based on the analysis of the intrinsic reactivity of the molecule in question through easily calculated chemical properties, such as OH bond dissociation enthalpies (BDEs), proton affinities (PAs) and ionization potentials (IPs). The BDEs can be used to estimate the viability of the HAT mechanism, while PAs and IPs are descriptors related to the first and second steps of the SPLET mechanism, respectively.

Another computational approach to evaluate the direct antioxidant activity is based on DFT computations of reaction rate constants (for Equations 1–4), using a model free radical, such as the hydroperoxyl radical (HOO^.^). HOO^.^ is considered a good free radical model for the comparative analysis of molecules with potential antioxidant activity, due to its moderate reactivity as compared to other free radicals such as HO^.^ that react too fast with any potential antioxidant (and are thus unsuitable for meaningful comparisons) (Rose and Bode, 1993; Galano et al., 2011). Moreover, the HOO^.^ radical participates in both hydrogen atom and electron transfer reactions (Galano and Alvarez-Idaboy, 2011; León-Carmona et al., 2012).

As for the indirect antioxidant activity of **Q** and its oxidation products, we have approached it with a model widely studied experimentally and to a lesser extent theoretically. Nowadays it is well accepted that the nuclear transcription factor Nrf2 (nuclear factor erythroid 2-related factor 2) induces the expression of genes encoding for detoxifying enzymes, antioxidants and cytoprotective proteins. In the cytoplasm, Nrf2 is generally bound to its inhibitor called Kelch-like ECH-1-associated protein (Keap1), which serves as an adapter for the degradation of Nrf2. Many studies show that the Keap1/Nrf2 signaling pathway plays a crucial role in maintaining the balance of cellular redox homeostasis. Ji et al. (2015) observed the contribution of the Keap1/Nrf2 signaling pathway to the protection by **Q** against hepatotoxicity. These authors suggest, based on their molecular docking calculations, that quercetin may interact with Keap1 and occupy the binding site of Nrf2 (in the Keap1 protein), thus facilitating the dissociation of Keap1 and Nrf2, ultimately inducing the transcriptional activation of Nrf2. Based on these precedents, in this work, we carried out molecular docking calculations with **Q** and its oxidation products, in order to have an energetic criterion to judge the affinity of these compounds for the active site inside Keap1.

## Computational Methods

First, a conformational analysis of **Q**, **Fl**, and **Bf** was carried out. This was done by performing random rotations of the freely rotating bonds in these molecules in the range of 0° to 360°, generating 50 random structures for each system in this manner. Then, the generated structures were optimized at the M05-2X/6-31+G(d,p) level (Zhao et al., 2006), using the SMD solvation model (Marenich et al., 2009) with water as solvent to emulate the biological conditions. Subsequently, their vibrational frequencies were calculated to confirm them as true minima on their corresponding potential energy surfaces.

All reported chemical properties were computed at the M05-2X/6-31+G(d,p) level, including implicit solvent effects (water). To calculate the BDEs, the hydrogen atoms of the OH groups at positions 3, 5, 7, 3′, and 4′ for **Q** and **Bf**, and 2, 5, 7, 3′, 4′ for **Fl**, were considered, see Figure 1. Then, the BDEs were calculated as the enthalpy change at 298.15 K of the following reaction:

(5) $$\begin{array}{r} ArOH\to ArO^{\cdot }+H^{\cdot } \end{array}$$

PAs were calculated as the enthalpy change at 298.15 K of reaction (2) for all anions generated by deprotonating each of the OH groups at positions 3, 5, 7, 3′ and 4′ in **Q** and **Bf**, and 2, 5, 7, 3′, 4′ in **Fl**. In these calculations, the reported values for the enthalpy of the proton in vacuum (6.197 kJ·mol^−1^) (Klein et al., 2009) and the correction for the aqueous solvation enthalpy of the proton ΔH_solv_ = −1090 kJ·mol^−1^ (Atkins, 1998), were used.

IPs were calculated using three different approaches: Koopmans' theorem (IP_K_), vertical (IP_V_), and adiabatic (IP_A_). IP_K_ is defined as the negative of the HOMO energy. IP_V_ and IP_A_ correspond to the difference in energy of the molecule with N and N-1 electrons. For the IP_V_ approach, the N-1 electron system energy is computed at the geometry of the N electron system, whereas for IP_A_, the geometry of both systems, with N and N-1 electrons, correspond to their ground states.

The kinetics of the proposed mechanisms was evaluated using the hydroperoxyl radical (HOO^.^) as a model of peroxyl radicals in general (ROO^.^). For the HAT mechanism, the transition states were optimized in the different positions where **Q** and its derivatives can react with HOO^.^, i.e., at the OH groups. Then, the rate constants were determined using transition state theory (Truhlar et al., 1983):

(6) $$\begin{array}{r} k\left(T\right)=\kappa \left(T\right)\frac{k_{B}T}{h}e^{-\frac{{\Delta G}^{‡,1M}}{RT}} \end{array}$$

where κ*(T)* is the transmission coefficient, calculated using the Eckart approach (Eckart, 1930), which accounts for the quantum tunneling effect, *k*_*B*_ is the Boltzmann constant, *T* is the temperature, *h* is Planck's constant, *R* is the universal gas constant and ΔG^‡, 1M^ is the Gibbs free energy of activation under standard conditions (1.00 M concentration for solutes). At 298.15K, ΔG^‡, 1M^ is calculated from the Gibbs free energy of activation at standard pressure ΔG^‡, 1atm^ as:

(7) $$\begin{array}{r} \Delta G^{‡,1M}=\Delta G^{‡,1atm}-RT\ln\left(24.5\right) \end{array}$$

The corresponding ΔG^‡^ values for electron transfer reactions were calculated using Marcus theory (Marcus, 1993):

(8) $$\begin{array}{r} \Delta G^{‡}=\frac{\lambda }{4}{\left(1+\frac{\Delta G}{\lambda }\right)}^{2} \end{array}$$

where ΔG is the Gibbs free energy of reaction and λ is the reorganization energy, which can be estimated as the difference between ΔE (non-adiabatic energy difference between the reactants and products) and ΔG (Nelsen et al., 2006).

Additionally, when the rate constants are equal to or greater than the diffusion limit, they lack physical meaning. In such cases, the kinetics of the reaction is controlled by the rate at which the reactants can diffuse through the solvent. For this reason, the apparent rate constants, *k*_*app*_, were calculated using the Collins-Kimball theory (Collins and Kimball, 1949):

(9) $$\begin{array}{r} k_{app}\left(T\right)=\frac{k_{d}k\left(T\right)}{k_{d}+k\left(T\right)} \end{array}$$

where *k*_*d*_ is the steady-state Smoluchowski rate constant for an irreversible bimolecular diffusion-controlled reaction (Smoluchowski, 1918). Geometry optimizations and vibrational frequencies were computed with the Gaussian 16 package (Frisch et al., 2016), and the rate constants were calculated using the Eyringpy program (Dzib et al., 2019).

Molecular docking analyses were performed to study the possible binding modes of **Q** and its oxidation products to Keap1 as potential inhibitors. The binding site of human Keap1 inhibitors has been characterized based on structural information derived from several cocrystals (PDB code: 4IN4, 4IQK, 4L7B, 4L7C, 4L7D, 4N1B, 3VNG, 3VNH). AutoDock (v 4.2.1) and AutoDock Vina (v 1.0.2) (Trott and Olson, 2010) were used for all dockings in this study. The ligand files were prepared using the AutoDockTools package (Sanner, 1999) provided by AutoDock by accepting all rotatable bonds. The cocrystal structure of Keap1 (Jnoff et al., 2014) (PDB Code: 4L7B) was downloaded from the Protein Data Bank (Berman et al., 2000). The Keap1 was treated with the Schrödinger's Protein Preparation Wizard (Madhavi Sastry et al., 2013); polar hydrogen atoms were added, nonpolar hydrogen atoms were merged, and charges were assigned. Docking was treated as rigid and carried out using the empirical free energy function and the Lamarckian Genetic Algorithm provided by AutoDock Vina (Morris et al., 1998). The grid map dimensions were 25 × 25 × 25 points, with 0.375 Å spacing between grid points, making the binding pocket of Keap1 the center of the cube. All other parameters were set as the default defined by AutoDock Vina. Dockings were repeated 20 times with space search exhaustiveness set to 20. The best interaction binding energy (kcal·mol^−1^) was selected for evaluation.

To reveal possible non-covalent Keap1-metabolite interactions, such as hydrogen bonds, steric repulsion, and van der Waals interactions, the non-covalent interaction index (NCI)(Johnson et al., 2010; Contreras-García et al., 2011) was used. The NCI is based on the electron density (ρ), its derivatives and the reduced density gradient (*s*). The reduced density gradient is given by:

(10) $$\begin{array}{r} s=\frac{1}{2{\left({3\pi }^{2}\right)}^{1/3}}\;\frac{\nabla \rho }{\rho^{4/3}} \end{array}$$

In this methodology, non-covalent interactions appear at low electron densities and reduced density gradient values, so the isosurface of the reduced density gradient is represented at low values. In this way, weak interactions can be visualized as closed domains in the molecular space located in the regions where the interactions occur. However, attractive and repulsive interactions appear in the same regions; therefore, to distinguish the attractive and repulsive interactions, the sign of the second eigenvalue of the electron density Hessian matrix (λ_2_) is used. A positive or negative sign is related to an inward or outward electron gradient flow, respectively, for a specific spatial region, so that attractive and repulsive interactions appear at negative and positive λ_2_ values, respectively. In this work, the promolecular densities (ρ^*pro*^), computed as the sum of all atomic contributions, were used. The NCI was calculated using the NCIPLOT program (Contreras-García et al., 2011).

## Results and Discussion

**Figure 2:**
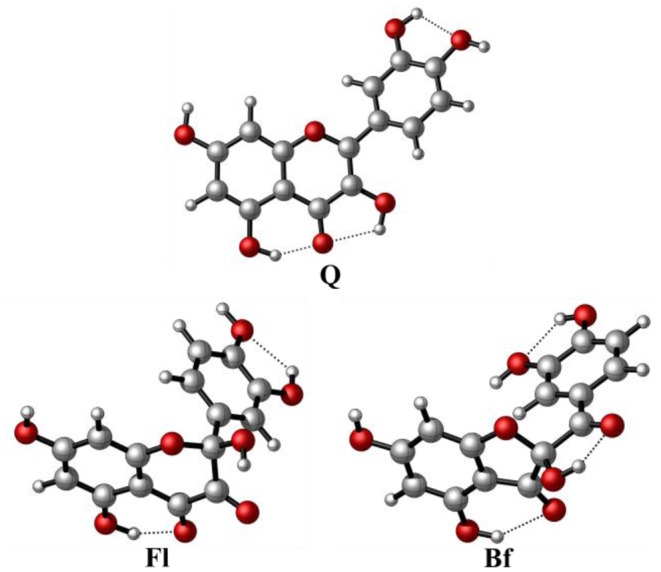
Structure of the most stable conformers of quercetin and its main oxidized derivatives at the M05-2X/6-31+G(d,p) level.

The most stable conformers of **Q**, **Fl**, and **Bf**, optimized at the M05-2X/6-31+G(d,p) level, in solvent (water) using the SMD continuum model, are shown in Figure 2. The Cartesian coordinates of the lowest energy conformers and their corresponding relative energies are reported in the Supplementary Material. In the case of Qox, **Bf** is 5.7 kcal·mol^−1^ more stable than **Fl**, which is mainly attributed to the formation of an additional H-bond in **Bf**.

Table 1 shows the calculated OH BDEs and PAs. The OH corresponding to each of the BDEs is identified according to the carbon numeration in Figure 1. The PAs were calculated for all anions generated by the deprotonation of each of the OH groups.

**Table 1:** OH bond dissociation enthalpies (BDEs) for all phenolic hydrogens and proton affinities (PAs) for all possible conjugate bases of **Q**, **Fl**, and **Bf**.

**System**	**OH position**	**BDE (kcal·mol^**−1**^)**	**PA (kcal·mol^**−1**^)**
**Q**	3	84.2	26.4
	5	96.3	28.3
	7	95.8	23.5
	3′	87.3	28.6
	4′	83.8	23.2
**Fl**	2	108.2	25.5
	5	111.2	24.1
	7	106.9	19.1
	3′	87.7	28.1
	4′	86.3	24.1
**Bf**	3	114.5	26.4
	5	112.1	20.5
	7	112.0	20.5
	3′	87.4	23.8
	4′	90.3	22.6

In general, the PA values are significantly lower than the BDE values in all systems, so from a thermodynamic point of view, the first stage of the SPLET mechanism (antioxidant deprotonation) seems to be the preferred pathway in all three systems. The data depicted in Table 1 show that: on the one hand, according to the BDE values, the HAT mechanism would be more favored in **Q** than in Qox, with the OH group at position 4′, located on the B ring of **Q**, being the most prone to transfer hydrogen. In Qox, the most active OH groups to transfer hydrogen via HAT mechanism are those located on the B ring. On the other hand, the lowest PA values correspond to the anions generated when deprotonating the OH group at position 7-OH of **Fl** (19.1 kcal·mol^−1^) and at position 5-OH or 7-OH of **Bf**, with the same value (20.5 kcal·mol^−1^), while in **Q**, the lowest PA value corresponds to the anion obtained when deprotonating the OH at position 4′-OH (23.2 kcal·mol^−1^). This not only indicates that the first step of the SPLET mechanism is favored in the Qox species, but also that at the same pH value (i.e., pH = 7.4), there will be a higher concentration of the conjugate base of **Fl** and **Bf** than that of **Q**.

To verify this initial assessment, the corresponding pathways of both HAT and SPLET mechanisms were evaluated, using the model radical (HOO^.^). Table 2 shows the Gibbs free energies of reaction (ΔG) for the reaction of **Q**, **Fl**, and **Bf** with HOO^.^ at 298.15 K via the HAT mechanism. The different values correspond to the transfer of one hydrogen atom from the indicated position; see Figure 1. According to the free reaction energies, the only favorable reaction paths are those where radical HOO^.^ attacks **Q** at the 3-OH, 3′-OH and 4′-OH positions, **Fl** at positions 3′-OH and 4′-OH and **Bf** at position 3′-OH, for which the Gibbs free energies of activation (ΔG^‡, 1M^) and the corresponding apparent rate constants were calculated and reported in Table 3. The reaction free energy profiles and the structure of the corresponding transition states are shown in the Supplementary Figures 1–6. Only at position 4′-OH of **Q**, is the *k*_*app*_ value (1.2 × 10^3^ L·mol^−1^·s^−1^) around the same order of magnitude as the rate constant of the reaction of HOO^.^ with polyunsaturated fatty acids (Itagaki et al., 2009). This is important to consider, since the antioxidant must react faster with the free radical than the biomolecules to be protected (e.g., the polyunsaturated fatty acids). Interestingly, the favorable reaction paths coincide with the lowest BDE values.

**Table 2:** Gibbs free energies of reaction for the hydrogen atom transfer reaction of HOO^.^ with **Q**, **Fl**, and **Bf** at the phenolic positions.

**System**	**OH position**	**ΔG (kcal·mol^**−1**^)**
**Q**	3	−4.2
	5	7.9
	7	7.5
	3′	−1.0
	4′	−4.5
**Fl**	2	19.6
	5	22.2
	7	18.6
	3′	−1.0
	4′	−2.3
**Bf**	3	26.8
	5	24.4
	7	24.2
	3′	−0.3
	4′	2.6

**Table 3:** Gibbs free energies of activation and apparent rate constants for the favorable hydrogen atom transfer reaction of HOO^.^ with **Q**, **Fl**, and **Bf**.

**System**	**OH position**	**ΔG^‡^^**, 1M**^ (kcal·mol^**−1**^)**	**k_**app**_ (L·mol^−1^·s^−1^)**
**Q**	3	16.5	2.2 × 10^2^
	3′	38.5	3.1 × 10^−15^
	4′	16.2	1.2 × 10^3^
**Fl**	3′	24.1	1.7 × 10^−5^
	4′	21.0	4.5 × 10^0^
**Bf**	3′	18.7	4.2 × 10^1^

**Figure 3:**
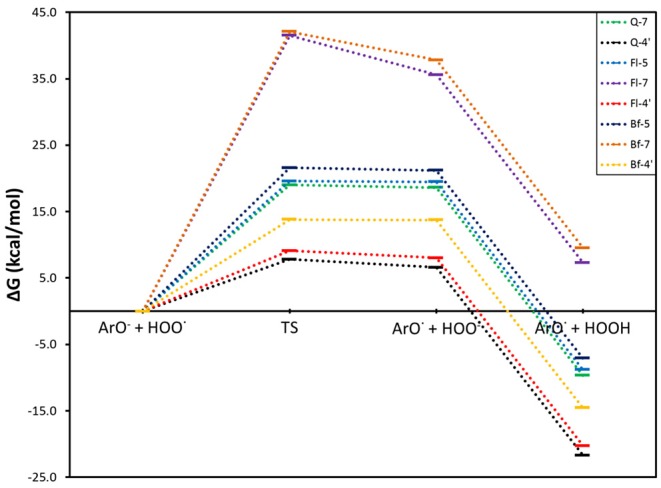
Gibbs free energy profile for the electron transfer reaction of the selected conjugate bases of **Q** and its oxidized derivatives with the HOO^.^ radical.

For the SPLET mechanism pathway, the conjugated bases of **Q**, **Fl**, and **Bf** were taken as the reagents, considering the lowest PA values previously obtained (Table 1), that is, the anions obtained by deprotonating **Q** at the 7-OH and 4′-OH positions and **Fl** and **Bf** at the 5-OH, 7-OH and 4′-OH positions. The corresponding reaction profiles are shown in Figure 3 and the corresponding Gibbs free energies of activation, ionization potentials (calculated using Koopmans' theorem (IP_K_), vertical (IP_V_) and adiabatic (IP_A_) approaches) and rate constants are reported in Table 4, where the conjugate bases are labeled according to the OH group from which a proton is removed.

**Table 4:** Ionization potentials of the selected conjugated bases of **Q**, **Fl**, and **Bf**, and Gibbs free energies of reaction and activation and rate constants for the electron transfer reaction of HOO^.^ with the selected conjugate bases.

**Conjugate base**	**IP_**K**_ (kcal·mol^**−1**^)**	**IP_**V**_ (kcal·mol^**−1**^)**	**IP_**A**_ (kcal·mol^**−1**^)**	**ΔG (kcal·mol^**−1**^)**	**ΔG^**‡, 1M**^ (kcal·mol^**−1**^)**	**k_app_ (L·mol^−1^·s^−1^)**
**Q-7**	154.8	125.5	122.8	−9.7	19.0	7.5 × 10^−2^
**Q-4′**	145.0	114.7	111.0	−21.7	7.8	1.2 × 10^7^
**Fl-5**	162.3	129.2	124.1	−8.8	19.6	2.7 × 10^−2^
**Fl-7**	166.8	144.6	138.6	7.3	41.5	2.3 × 10^−18^
**Fl-4′**	150.8	117.1	112.7	−20.3	9.1	1.4 × 10^6^
**Bf-5**	163.7	130.3	125.8	−7.1	21.6	9.3 × 10^−4^
**Bf-7**	164.8	150.3	142.0	9.5	42.1	8.7 × 10^−19^
**Bf-4′**	154.8	122.4	118.1	−14.6	13.8	4.8 × 10^2^

In this mechanism, the rate determining step is the electron transfer from the antioxidant conjugate base to the HOO^.^ radical. Subsequently, the HOO^−^ anion is rapidly protonated to form HOOH in a highly exergonic process. From Figure 3, it can be concluded that the SPLET mechanism is more favorable for the conjugate bases obtained by deprotonating the three systems at the 4′-OH position. In addition, these conjugate bases have the lowest activation energies for electron transfer and therefore the highest rate constants. The conjugate bases of **Q** at the 7-OH position and **Fl** and **Bf** at the 5-OH position also have favorable reaction pathways, but their activation energies of around 20 kcal·mol^−1^ make this process too slow to be competitive with the conjugate bases at the 4′-OH position, see Table 4. The conjugate bases of **Fl** and **Bf** at the 7-OH position do not present a favorable reaction pathway (ΔG > 0). Interestingly, these trends are consistent with the calculated ionization potentials. Note that the electron transfer rate constants of the conjugate bases of **Q** and **Fl** at the 4′-OH position are at least three orders of magnitude higher than the rate constants calculated for the HAT mechanism, so it can be deduced that the direct antioxidant activity of **Q** and its oxidized derivatives arises mainly via SPLET mechanism, **Q-4**′ being the conjugate base with the highest antioxidant activity, surpassing the electron transfer rate constant of **Fl-4**′ by an order of magnitude and that of **Bf-4**′ by five orders of magnitude.

However, it is important to point out that, as mentioned before, the PA values are lower in Qox, so although the conjugate bases of **Fl** and **Bf** react more slowly via the SPLET mechanism, it would be expected that these are present in a greater proportion than the conjugate base of **Q**, so a balance among all the factors in play would be expected, which will depend on the pH and the acidity of Qox, for which there is no reported experimental value, but for **Q** (pK_a_ = 8.45) (Musialik et al., 2009).

**Figure 4:**
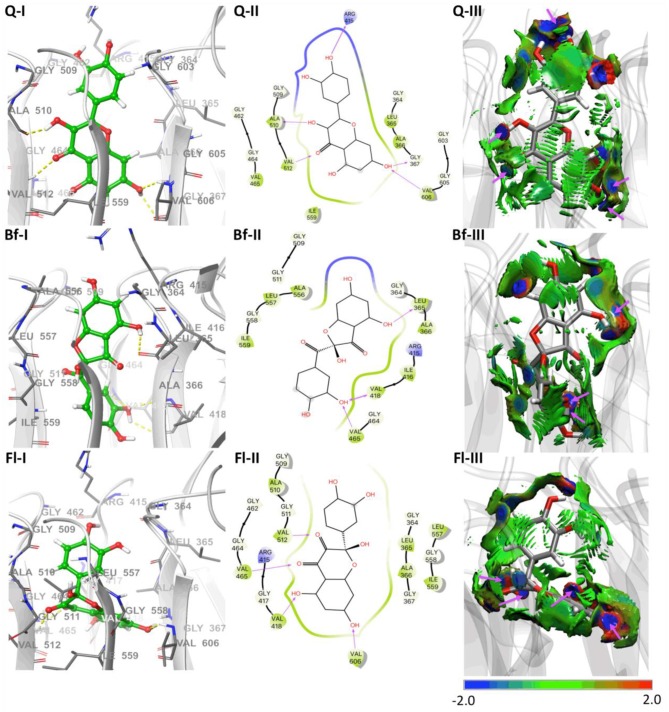
Molecular docking results of **Q** and Qox on Keap1. (**I**) Representative amino acid residues surrounding **Q** or Qox in the binding pocket of Keap1. (**II**) Two-dimensional interaction map of **Q** or Qox and human Keap1. The arrows indicate potential interactions between amino acid residues and **Q** or Qox. (**III**) NCIPLOT isosurface gradient (0.6 au) of **Q** or Qox on the structure of Keap1. The surfaces are colored on a blue-green-red scale according to the strength and type of interaction. Blue indicates strong attractive interactions, green indicates weak van der Waals interactions, and red indicates a strong nonbonded overlap.

Molecular docking calculations, which were performed to estimate the possible binding sites of metabolites in the Keap1 kelch domain, show that **Q**, **Bf**, and **Fl** should act as inhibitors of the protein-protein interaction between Keap1 and Nrf2. This inhibitory capability would favor the release of Nrf2, which in turn would favor the quenching of radical species. For this reason, in a very simplified approach, the metabolite that has a better affinity for Keap1 should act as a better indirect antioxidant. The coupling modes of **Q**, **Bf**, and **Fl** at the binding site of the human Keap1 is illustrated in the three-dimensional and two-dimensional interaction maps of Figure 4. It can be seen that the binding site of **Q** in the Keap1 coincides with those identified and reported in previous work (Jnoff et al., 2014; Gacesa et al., 2015; Ji et al., 2015; Li et al., 2017; Zhuang et al., 2017; Yang et al., 2018). Besides, the three metabolites are embedded within the protein pocket, through hydrophobic and electrostatic interactions with the Gly364, Leu365, Ala366, Gly367, Arg415, Ile416, Gly417, Val418, Gly462, Gly464, Val465, Gly509, Gly509, Ala510, Gly511, Val512, Ala556, Ala556, Leu557, Gly558, Ile559, Gly603, Gly605, and Val606 residues. In the case of **Q**, it presents five hydrogen bonding interactions: (i) between hydrogen at position 4′-OH and Arg415, (ii) between hydrogen at position 3-OH and Ala510, (iii) between hydrogen at position 7-OH and Gly367 (iv) between oxygen at position 7-OH and Val606 and, finally, (v) between the **Q** carbonyl group and Val512. In the case of the Qox, **Bf**, and **Fl** have three and four hydrogen-bonding interactions, respectively. For **Bf**: (i) between hydrogen at position 5-OH and Leu365, (ii) between hydrogen at position 3′-OH and Val418, (iii) between oxygen at position 3′-OH and Val465. For **Fl**: (i) between the carbonyl group at position 3 with Val512, (ii) between the carbonyl group at position 4 with Val465, (iii) between hydrogen at position 5-OH and Val418 and, (iv) between hydrogen at position 7-OH and Val606. It can therefore be concluded that hydrogen bonds, as well as hydrophobic forces, dominate the interactions in these complexes. Finally, the estimated interaction Gibbs free energies (ΔG) are −9.9, −9.2, and −8.9 kcal·mol^−1^, for the **Bf**-Keap1, **Q**-Keap1, and **Fl**-Keap1 complexes, respectively. Interestingly, the best Keap1-metabolite interaction energy is that of **Bf**, which is identified as the most thermodynamically stable oxidized isomer by our quantum chemical calculations. Finally, the NCIPLOT analysis identified all the hydrogen bonding interactions in total agreement with the docking analysis, providing a qualitative confirmation of these interaction but now from topological and visual analysis of a scalar field related to the electron density. Therefore, these results are broadly in line with experimental evidence, which shows that Qox outperform **Q**'s antioxidant activity only when tested in cellular assays. These results suggest that the better antioxidant character of these oxidized species is due to indirect mechanisms.

## Conclusions

In this work, the activity of **Q** and its main oxidized species (Qox) as both direct and indirect antioxidants is compared through a theoretical analysis. The computed proton affinities (PA) are significantly lower than the computed hydrogen bond dissociation energy (BDE) values in all studied cases, suggesting that the first stage of the SPLET mechanism is the preferred pathway for **Q** and Qox, being more favored in the Qox species. On the other hand, the computed electron transfer rate constants of the conjugate bases of **Q** and **Fl** at the 4′-OH position are at least three orders of magnitude higher than the rate constants calculated for the HAT mechanism, supporting also that SPLET is the preferred mechanism. Thus, considering the SPLET mechanism, **Q-4**′ should be the conjugate base with the highest antioxidant activity, surpassing the electron transfer rate constant of **Fl-4**′ by an order of magnitude and that of **Bf-4**′ by five orders of magnitude. However, PA values are lower in the oxidized derivatives of **Q**, so although the conjugate bases of **Fl** and **Bf** react more slowly via the SPLET mechanism, it would be expected that these are present in a more significant proportion than the conjugate base of **Q**, Therefore, it is not possible to definitively conclude, on the basis of our calculations, whether **Q** or Qox have greater direct antioxidant activity. Molecular docking calculations predict that both **Q** and Qox species present a stabilizing interaction within the Keap1 kelch domain, suggesting their potential activity as inhibitors of the protein-protein interaction between Keap1 and Nrf2. Our results show that the most thermodynamically stable metabolite (**Bf**), according to our quantum chemical calculations, presents the best interaction energy. Moreover, the NCIPLOT analysis identified all the hydrogen bonding interactions, providing a qualitative confirmation of these interactions from a quantum chemical perspective. All our computational results support the experimental evidence that Qox outperform quercetin's antioxidant activity only when tested in cellular assays. However, more computational work will have to be done to clarify the mechanisms involved in the indirect antioxidant activity of these species.

## Data Availability Statement

All datasets generated for this study are included in the article/Supplementary Material.

## Author Contributions

AV-E, BC, LR, and WT contributed to the conception and design of the study. AV-E and OY preformed the theoretical calculations. AV-E, EO, and OY organized the database. AV-E, BC, and WT wrote the first draft of the manuscript. EO, CA, LR, OG-B, and OY wrote sections of the manuscript. All authors contributed to manuscript revision, read, and approved the submitted version.

### Conflict of Interest

The authors declare that the research was conducted in the absence of any commercial or financial relationships that could be construed as a potential conflict of interest.
