# Supplementary material for: Theoretical Study of the Antioxidant Activity of Quercetin Oxidation Products
Source: Front Chem. 2019 Nov 27;7:818. doi: 10.3389/fchem.2019.00818 (PMC6890856; doi:10.3389/fchem.2019.00818)
Supplement: Supplementary file 1 [file Data_Sheet_1.PDF]

Supplementary Material

Theoretical Study of the Antioxidant Activity of Quercetin Oxidation Products

Alejandro Vasquez-Espinal[1], Osvaldo Yañez[1], Edison Osorio[2], Carlos Areche[3], Olimpo García[2], Lina María Ruiz[4], Bruce K. Cassels[3*] and William Tiznado[1*]

[1] Computational and Theoretical Chemistry Group, Departamento de Ciencias Químicas, Facultad de Ciencias Exactas, Universidad Andres Bello, República 498, Santiago, Chile

[2] Facultad de Ciencias Naturales y Matemáticas, Universidad de Ibagué, Carrera 22 calle 67, Ibagué, Colombia

[3] Departamento de Química, Facultad de Ciencias, Universidad de Chile, Las Palmeras 3425, Ñuñoa, Santiago, Chile

[4] Instituto de Ciencias Biomédicas, Facultad Ciencias de la Salud, Universidad Autónoma de Chile, Llano Subercaseaux 2801, San Miguel, Santiago, Chile

1 Supplementary Data

Cartesian coordinates of the lowest energy conformers of Quercetin and its main oxidized products and their corresponding relative energies at the M05-2X/6-31+G(d,p) level including the zero-point energy (ZPE) correction.

Q					
C	1.622730000	0.708915000	-0.101299000	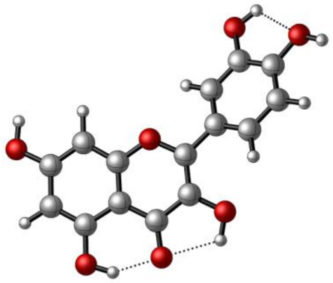	ΔE = 0.0 kcal/mol
C	2.368305000	-0.470304000	0.026552000		
C	2.212622000	1.961315000	-0.201683000		
C	3.783664000	-0.362807000	0.057369000		
C	3.601836000	2.017019000	-0.171222000		
C	4.397115000	0.869063000	-0.042062000		
C	-0.423879000	-0.507401000	-0.042930000		
C	0.241618000	-1.685330000	0.091185000		
O	0.266854000	0.665672000	-0.138438000		
C	1.685866000	-1.727741000	0.132925000		
C	-1.875374000	-0.304170000	-0.081035000		
C	-2.741649000	-1.292706000	-0.561310000		
C	-2.397174000	0.924672000	0.354151000		
C	-4.112874000	-1.057121000	-0.601125000		
C	-3.760758000	1.149425000	0.317761000		
C	-4.625297000	0.156461000	-0.161446000		
O	-5.955393000	0.473144000	-0.160753000		
O	-4.256494000	2.350622000	0.756043000		
O	2.268992000	-2.834106000	0.272169000		
O	-0.407347000	-2.876329000	0.227584000		
O	4.544901000	-1.475096000	0.183805000		
O	4.263665000	3.200532000	-0.265932000		
H	1.606784000	2.852465000	-0.300548000		
H	5.475425000	0.951322000	-0.020173000		
H	-2.355459000	-2.233979000	-0.922824000		
H	-1.750665000	1.706327000	0.730809000		
H	-4.792750000	-1.810845000	-0.980714000		
H	-6.482398000	-0.259090000	-0.508806000		
H	-5.220314000	2.351575000	0.666624000		
H	0.280204000	-3.552341000	0.358136000		
H	3.940206000	-2.248375000	0.247540000		
H	3.637040000	3.931683000	-0.360856000		
C	-1.639448000	0.713435000	0.131773000	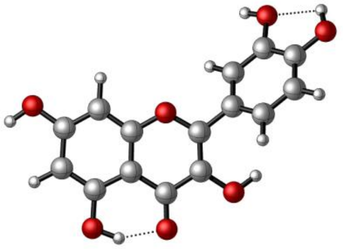	ΔE = 2.4 kcal/mol
C	-2.356268000	-0.479508000	-0.035539000		
C	-2.253988000	1.948607000	0.267519000		
C	-3.772763000	-0.398663000	-0.069472000		
C	-3.644286000	1.978432000	0.229845000		
C	-4.413465000	0.818335000	0.064152000		
C	0.417127000	-0.469168000	0.051049000		
C	-0.211916000	-1.655217000	-0.156533000		
O	-0.280573000	0.695424000	0.176801000		
C	-1.658612000	-1.729427000	-0.193990000		
C	1.870061000	-0.262026000	0.131182000		
C	2.680584000	-1.128232000	0.870333000		
C	2.438534000	0.838812000	-0.526549000		
C	4.053271000	-0.905763000	0.941641000		
C	3.803890000	1.050590000	-0.455079000		
C	4.617204000	0.175359000	0.279839000		
O	5.965577000	0.380455000	0.354839000		
O	4.450869000	2.087470000	-1.074628000		
O	-2.252169000	-2.821255000	-0.368586000		
O	0.425193000	-2.848196000	-0.361560000		
O	-4.510971000	-1.519458000	-0.235064000		
O	-4.234512000	3.196335000	0.362151000		
H	-1.673244000	2.850994000	0.397502000		
H	-5.494740000	0.869626000	0.037159000		
H	2.245241000	-1.956340000	1.415646000		
H	1.823066000	1.520450000	-1.102005000		

H	4.694732000	-1.561146000	1.516893000		
H	6.204244000	1.169051000	-0.154054000		
H	3.823802000	2.634091000	-1.567310000		
H	1.366216000	-2.710467000	-0.542855000		
H	-3.878923000	-2.273933000	-0.323905000		
H	-5.197252000	3.118080000	0.310283000		
C	-1.756896000	-0.701447000	0.059578000	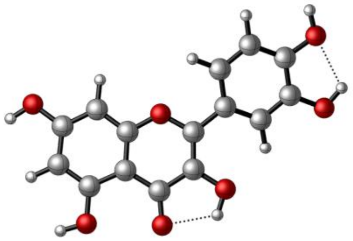	ΔE = 4.6 kcal/mol
C	-2.369793000	0.562471000	-0.022282000		
C	-2.467091000	-1.891009000	0.150794000		
C	-3.790095000	0.586105000	-0.006606000		
C	-3.850874000	-1.813165000	0.159740000		
C	-4.520058000	-0.584669000	0.080622000		
C	0.430757000	0.240208000	-0.035506000		
C	-0.089355000	1.488877000	-0.120256000		
O	-0.406162000	-0.833422000	0.059758000		
C	-1.525069000	1.732589000	-0.117362000		
C	1.843831000	-0.152914000	-0.059123000		
C	2.190887000	-1.449194000	-0.461267000		
C	2.853045000	0.743266000	0.325025000		
C	3.525036000	-1.840566000	-0.490096000		
C	4.178696000	0.346703000	0.293610000		
C	4.520805000	-0.947340000	-0.115779000		
O	5.855245000	-1.247773000	-0.112838000		
O	5.150409000	1.236840000	0.675943000		
O	-1.909156000	2.915831000	-0.210998000		
O	0.689874000	2.599890000	-0.247566000		
O	-4.409551000	1.790690000	-0.078504000		
O	-4.540472000	-2.980555000	0.249805000		
H	-1.951835000	-2.839224000	0.213291000		
H	-5.603752000	-0.551481000	0.089929000		
H	1.425768000	-2.151717000	-0.760831000		
H	2.622565000	1.743227000	0.662265000		
H	3.801731000	-2.839746000	-0.805841000		
H	5.996709000	-2.154999000	-0.415900000		
H	6.019432000	0.817818000	0.599222000		
H	0.066596000	3.346207000	-0.323953000		
H	-5.369014000	1.670632000	-0.037668000		
H	-5.492646000	-2.812234000	0.248115000		

**Fl**					
C	1.664148000	-0.077281000	0.850705000	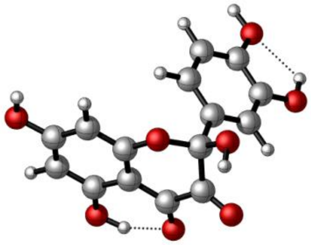	ΔE = 0.0 kcal/mol
C	1.755780000	0.558140000	-0.411529000		
C	2.487012000	-1.126653000	1.193804000		
C	2.725167000	0.075574000	-1.335823000		
C	3.427556000	-1.565580000	0.250738000		
C	3.553767000	-0.982300000	-1.010735000		
C	-0.420613000	1.017869000	1.386843000		
C	-0.033824000	2.111620000	0.389408000		
O	0.777076000	0.342596000	1.803550000		
C	0.934038000	1.687379000	-0.714668000		
C	-1.390619000	0.060578000	0.717545000		
C	-1.232384000	-1.318450000	0.821364000		
C	-2.487760000	0.598648000	0.037258000		
C	-2.175534000	-2.166438000	0.242043000		
C	-3.423246000	-0.247275000	-0.534111000		
C	-3.264782000	-1.635754000	-0.435596000		
O	2.858876000	0.648660000	-2.546730000		
O	4.257792000	-2.593150000	0.530186000		
O	0.965696000	2.347887000	-1.758150000		
O	-0.484891000	3.230036000	0.431092000		
O	-1.014633000	1.541753000	2.522963000		
O	-4.237399000	-2.388125000	-1.035558000		
O	-4.499009000	0.286059000	-1.192861000		
H	2.399163000	-1.603122000	2.161510000		
H	4.289065000	-1.345229000	-1.715506000		
H	-0.394359000	-1.743136000	1.356836000		
H	-2.636965000	1.669233000	-0.045466000		
H	-2.071264000	-3.242661000	0.315894000		
H	2.232965000	1.399472000	-2.616521000		

H	4.095616000	-2.931960000	1.422311000		
H	-0.495932000	2.293038000	2.851392000		
H	-4.054445000	-3.331874000	-0.932408000		
H	-5.064166000	-0.429959000	-1.517450000		
C	1.776523000	-0.729223000	0.111511000	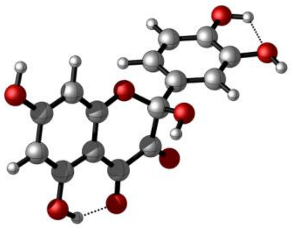	ΔE = 1.8 kcal/mol
C	2.357373000	0.537167000	-0.146582000		
C	2.545855000	-1.861167000	0.253057000		
C	3.775885000	0.613588000	-0.256911000		
C	3.938271000	-1.737385000	0.129073000		
C	4.563123000	-0.514261000	-0.115845000		
C	-0.422597000	0.179455000	0.565149000		
C	0.033179000	1.488838000	-0.108783000		
O	0.422250000	-0.902335000	0.175025000		
C	1.536765000	1.690289000	-0.323175000		
C	-1.825645000	-0.184806000	0.149577000		
C	-2.043478000	-0.880507000	-1.038057000		
C	-2.907438000	0.252004000	0.916198000		
C	-3.344112000	-1.155357000	-1.454291000		
C	-4.199850000	-0.025584000	0.497338000		
C	-4.422356000	-0.730183000	-0.690852000		
O	4.378828000	1.790090000	-0.506365000		
O	4.740871000	-2.815711000	0.247055000		
O	1.935663000	2.822104000	-0.618868000		
O	-0.742792000	2.366992000	-0.394260000		
O	-0.384540000	0.372785000	1.951611000		
O	-5.697506000	-1.005202000	-1.108002000		
O	-5.322524000	0.356246000	1.188574000		
H	2.084175000	-2.820511000	0.446902000		
H	5.638877000	-0.451546000	-0.204564000		
H	-1.207515000	-1.210525000	-1.640225000		
H	-2.756133000	0.806736000	1.833588000		
H	-3.532891000	-1.699599000	-2.371137000		
H	3.694622000	2.483439000	-0.617671000		
H	4.220719000	-3.617732000	0.399370000		
H	0.454809000	0.760600000	2.241525000		
H	-6.329924000	-0.628551000	-0.478940000		
H	-5.077718000	0.840750000	1.988557000		
C	1.659597000	-0.740005000	0.020204000	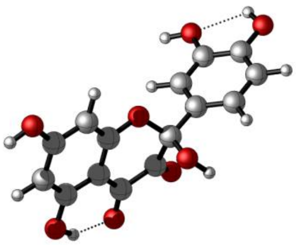	ΔE = 2.4 kcal/mol
C	2.318096000	0.503379000	-0.139009000		
C	2.347134000	-1.928152000	0.037897000		
C	3.732133000	0.496756000	-0.278245000		
C	3.743520000	-1.886631000	-0.106161000		
C	4.444641000	-0.691387000	-0.258987000		
C	-0.405051000	0.275574000	0.683699000		
C	0.057184000	1.586916000	0.026051000		
O	0.290059000	-0.816948000	0.103234000		
C	1.569902000	1.719471000	-0.182470000		
C	-1.878317000	0.010512000	0.508387000		
C	-2.733859000	-0.102051000	1.597630000		
C	-2.373021000	-0.126153000	-0.793781000		
C	-4.091473000	-0.353284000	1.391336000		
C	-3.718546000	-0.380144000	-0.993246000		
C	-4.584579000	-0.491876000	0.104097000		
O	4.410702000	1.647443000	-0.436826000		
O	4.380391000	-3.076835000	-0.085872000		
O	2.037638000	2.846242000	-0.377221000		
O	-0.681966000	2.505762000	-0.227143000		
O	0.026979000	0.347295000	2.021143000		
O	-5.917119000	-0.738172000	-0.088405000		
O	-4.295493000	-0.535751000	-2.227724000		
H	1.830860000	-2.870215000	0.158319000		
H	5.521486000	-0.685377000	-0.368750000		
H	-2.363226000	-0.006556000	2.609211000		
H	-1.717172000	-0.039200000	-1.654087000		
H	-4.773655000	-0.445846000	2.226973000		
H	3.773770000	2.392450000	-0.460968000		
H	5.336016000	-2.957465000	-0.184843000		
H	-0.411151000	1.078071000	2.483078000		
H	-6.097628000	-0.810615000	-1.036858000		
H	-3.632987000	-0.445096000	-2.925985000		

Bf					
C	-1.744544000	0.244193000	-0.882303000	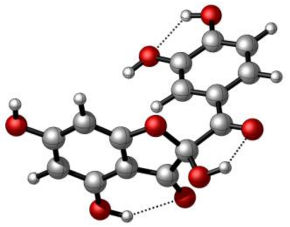	ΔE = 0.0 kcal/mol
C	-1.881233000	0.364999000	0.505602000		
C	-2.466379000	-0.651744000	-1.632275000		
C	-2.793643000	-0.448267000	1.197575000		
C	-3.372128000	-1.449086000	-0.908145000		
C	-3.548218000	-1.361391000	0.481531000		
O	-0.784733000	1.086204000	-1.388538000		
C	-0.312531000	1.931598000	-0.322603000		
C	-0.949920000	1.344725000	0.963877000		
C	1.237790000	1.963343000	-0.381799000		
C	2.046471000	0.751867000	-0.216183000		
C	3.703340000	-1.470291000	0.073204000		
C	4.261378000	-0.220394000	-0.179493000		
C	2.311305000	-1.611501000	0.183277000		
C	3.438019000	0.884711000	-0.323550000		
C	1.487705000	-0.511671000	0.040159000		
O	1.730146000	3.062806000	-0.595071000		
O	-0.693542000	1.737085000	2.094928000		
O	4.517610000	-2.546743000	0.210201000		
O	-0.809562000	3.214658000	-0.464658000		
O	-4.138861000	-2.366525000	-1.540685000		
O	-2.936221000	-0.350816000	2.536340000		
O	1.861792000	-2.881151000	0.431457000		
H	-2.353399000	-0.750647000	-2.703650000		
H	-4.262573000	-2.005269000	0.976150000		
H	5.337345000	-0.134496000	-0.259575000		
H	3.867683000	1.857346000	-0.520563000		
H	0.422143000	-0.673521000	0.132004000		
H	3.990953000	-3.338570000	0.397093000		
H	-0.079064000	3.775495000	-0.785887000		
H	-3.957220000	-2.376236000	-2.491303000		
H	-2.340963000	0.332048000	2.887039000		
H	0.897602000	-2.896835000	0.503658000		
C	2.523936000	-0.659149000	0.550537000	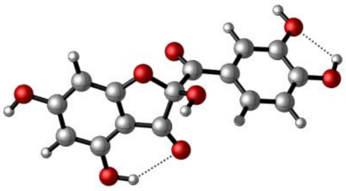	ΔE = 1.3 kcal/mol
C	2.362701000	0.596711000	-0.049574000		
C	3.687147000	-1.384848000	0.473675000		
C	3.430718000	1.182402000	-0.748789000		
C	4.728941000	-0.776781000	-0.246586000		
C	4.623141000	0.484468000	-0.853878000		
O	1.405699000	-1.061822000	1.226809000		
C	0.357217000	-0.108142000	1.010365000		
C	1.043394000	1.057514000	0.231897000		
C	-0.706359000	-0.780911000	0.104725000		
C	-2.101741000	-0.308604000	0.092958000		
C	-4.771481000	0.467427000	-0.098042000		
C	-3.847052000	1.311279000	0.504503000		
C	-4.361692000	-0.773847000	-0.614025000		
C	-2.515826000	0.924925000	0.610664000		
C	-3.039191000	-1.153777000	-0.528827000		
O	-0.323477000	-1.697463000	-0.606523000		
O	0.493245000	2.117959000	-0.038863000		
O	-6.072419000	0.850197000	-0.184648000		
O	-0.206392000	0.265742000	2.214863000		
O	5.880361000	-1.482527000	-0.328863000		
O	3.314971000	2.399776000	-1.320726000		
O	-5.351317000	-1.531985000	-1.184520000		
H	3.814066000	-2.353692000	0.934904000		
H	5.461559000	0.905215000	-1.394326000		
H	-4.183015000	2.265837000	0.888521000		
H	-1.810503000	1.593520000	1.079092000		
H	-2.729584000	-2.109362000	-0.934655000		
H	-6.586901000	0.159750000	-0.629329000		
H	0.438214000	0.769938000	2.734489000		
H	6.544568000	-0.996680000	-0.837459000		
H	2.432328000	2.768118000	-1.155460000		
H	-4.989223000	-2.353664000	-1.543638000		

C	-1.773457000	0.294866000	-0.847400000	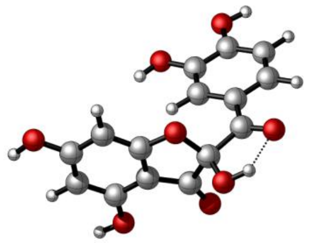	ΔE = 1.6 kcal/mol
C	-1.847069000	0.364384000	0.552055000		
C	-2.544771000	-0.545317000	-1.611479000		
C	-2.760681000	-0.458961000	1.232609000		
C	-3.449145000	-1.347279000	-0.897937000		
C	-3.568107000	-1.316990000	0.499635000		
O	-0.815578000	1.129577000	-1.370798000		
C	-0.289192000	1.933970000	-0.304856000		
C	-0.870460000	1.316894000	0.995308000		
C	1.257585000	1.948365000	-0.411055000		
C	2.056975000	0.728916000	-0.251041000		
C	3.691017000	-1.507280000	0.082873000		
C	4.259731000	-0.241766000	-0.040176000		
C	2.296201000	-1.659297000	0.032708000		
C	3.449809000	0.870436000	-0.208834000		
C	1.487283000	-0.549575000	-0.128343000		
O	1.759892000	3.043124000	-0.625679000		
O	-0.526347000	1.686308000	2.107828000		
O	4.426143000	-2.635072000	0.252673000		
O	-0.773318000	3.228169000	-0.388756000		
O	-4.222381000	-2.174439000	-1.637936000		
O	-2.802190000	-0.369258000	2.576932000		
O	1.811143000	-2.934140000	0.148052000		
H	-2.474671000	-0.595693000	-2.688576000		
H	-4.284072000	-1.960451000	0.997503000		
H	5.338005000	-0.143667000	0.002304000		
H	3.892381000	1.852915000	-0.299066000		
H	0.419594000	-0.716932000	-0.169775000		
H	5.367785000	-2.414612000	0.283919000		
H	-0.053452000	3.785634000	-0.737845000		
H	-4.822083000	-2.684842000	-1.075308000		
H	-3.463011000	-0.975957000	2.941487000		
H	0.846294000	-2.928734000	0.082435000		

2 Supplementary Figures

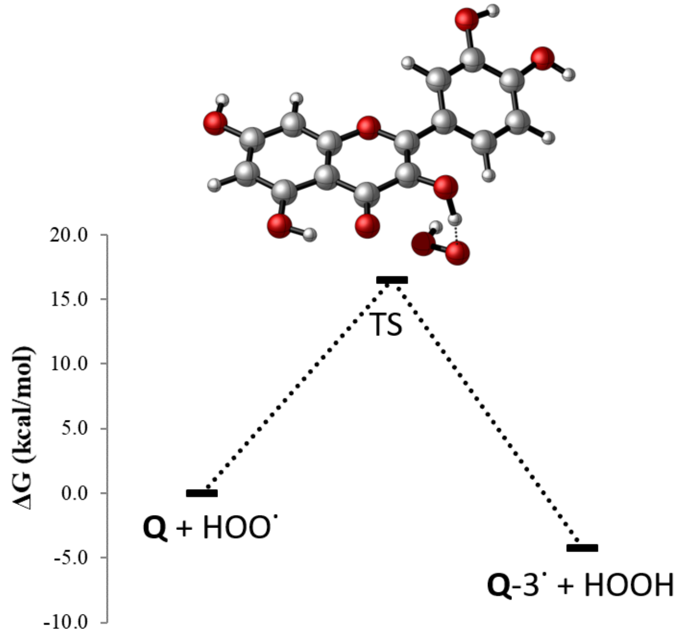

Supplementary Figure 1. Gibbs free energy profile of the hydrogen atom transfer reaction between **Q** and HOO˙ at the 3-OH position. The structure of the corresponding transition state is shown at the top.

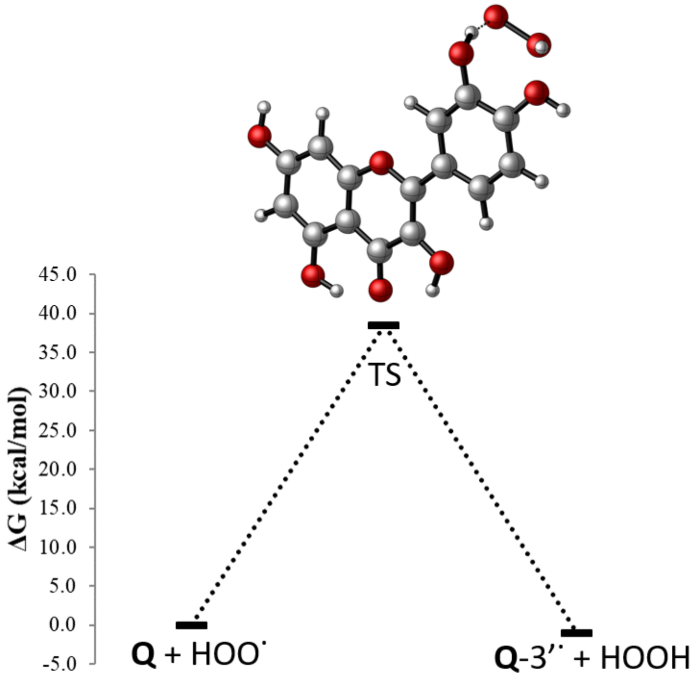

Supplementary Figure 2. Gibbs free energy profile of the hydrogen atom transfer reaction between **Q** and HOO˙ at the 3'-OH position. The structure of the corresponding transition state is shown at the top.

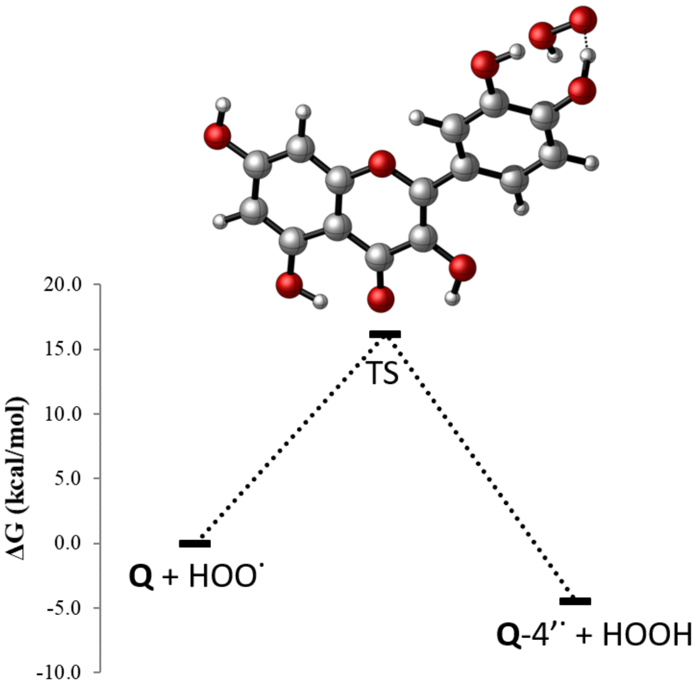

Supplementary Figure 3. Gibbs free energy profile of the hydrogen atom transfer reaction between **Q** and HOO˙ at the 4'-OH position. The structure of the corresponding transition state is shown at the top.

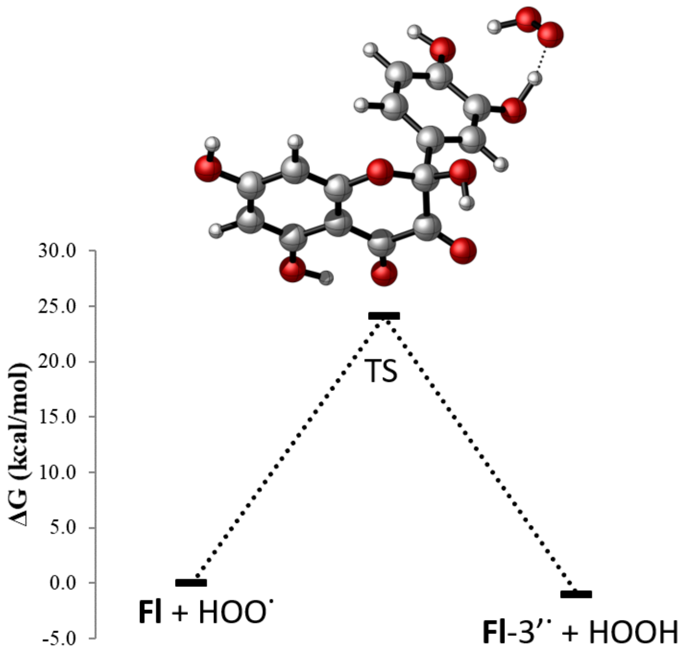

Supplementary Figure 4. Gibbs free energy profile of the hydrogen atom transfer reaction between **Fl** and HOO˙ at the 3'-OH position. The structure of the corresponding transition state is shown at the top.

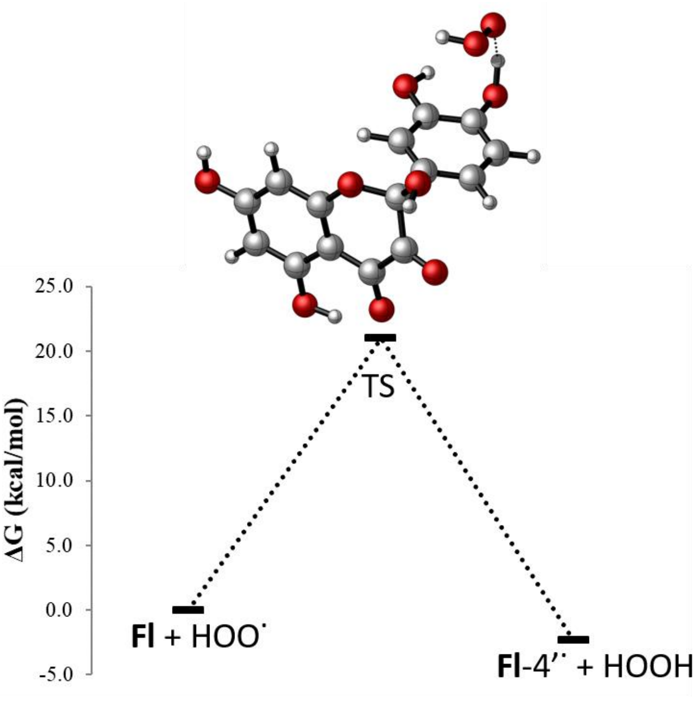

Supplementary Figure 5. Gibbs free energy profile of the hydrogen atom transfer reaction between **Fl** and HOO˙ at the 4'-OH position. The structure of the corresponding transition state is shown at the top.

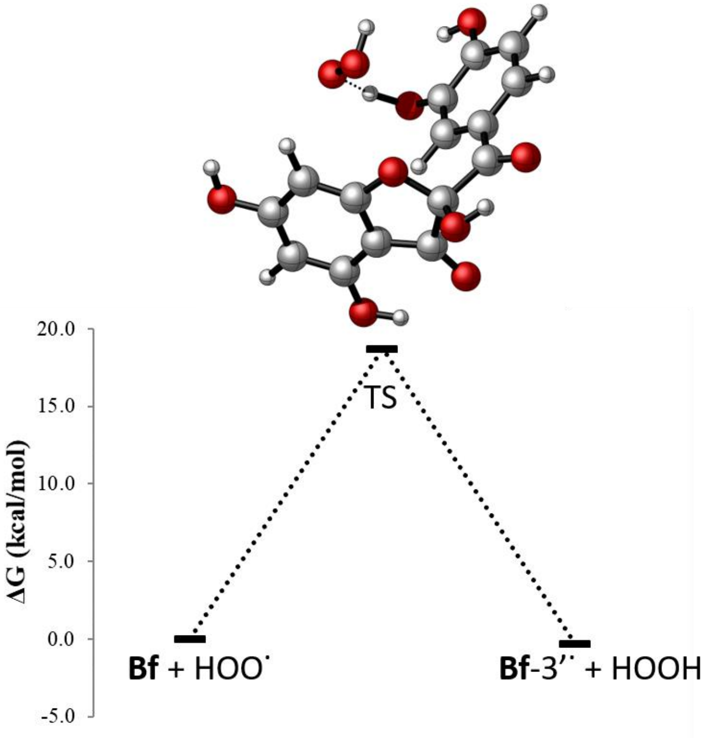

Supplementary Figure 6. Gibbs free energy profile of the hydrogen atom transfer reaction between **Bf** and HOO˙ at the 3'-OH position. The structure of the corresponding transition state is shown at the top.
